# The Role of p53 Family in Cancer

**DOI:** 10.3390/cancers14030823

**Published:** 2022-02-06

**Authors:** Joanna E. Zawacka-Pankau

**Affiliations:** Center for Hematology and Regenerative Medicine, Department of Medicine, Huddinge, Karolinska Institutet, 141 86 Stockholm, Sweden; Joanna.zawacka-pankau@ki.se

This Special Issue covers a broad topic on the role of the p53 protein family in cancer. The p53 protein is a critical sensor of cellular stress that has evolved in multicellular organisms and is a pivotal tumor suppressor in humans. It is widely recognized as the ‘guardian of the genome’ due to its ability to repair DNA damage when circumstances allow or to direct the affected cell to apoptosis; permanent cell elimination. This is how p53 prevents multicellular organisms from accumulating mutations and, in consequence, from developing cancer. 

p53 belongs to a family of proteins which includes p73 and p63. All p53 protein family members share the ability to induce apoptosis due to UV-induced DNA damage, through transcription-dependent or -independent functions. This is likely due to the fact that the first common ancestor protein for the three protein family members, p63/p73-like protein, found in the sea anemone, protects the germ-line gametes from UV damage [1]. In humans, p63, the most ancient of all three family members, retains the protective function of gametes and is responsible for the quality control in oocytes [2] and in sperm cells. *TP*73 and *TP*63 are ancestors of *TP*53 and apart from recognizing classical p53 promoters, they regulate the genes involved in neuronal development, inflammation, multiciliogenesis or epithelial tissue development. 

**Figure 1:**
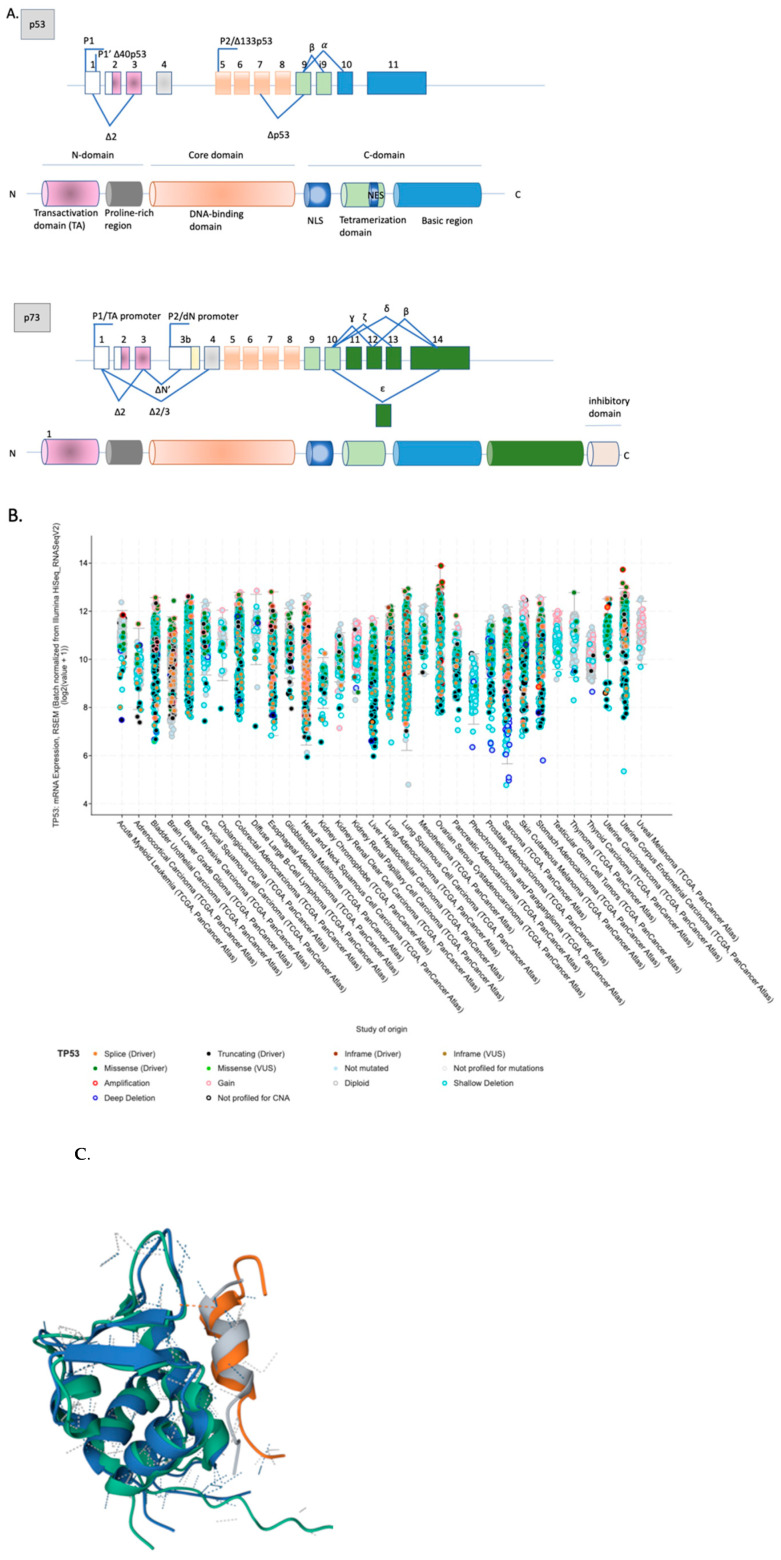
The structure of the p53 protein family. (**A**) Schemes of p53 and p73 isoforms adapted from [6]; (**B**) alignment of Np53 (grey) and Np73 (orange) in complex with MDM2 (cyan and blue) (PDB ID 1YCR and PDB ID 2MPS, respectively; dotted lines represent non-covalent interactions); (**C**) expression of *TP*53 mutations across 32 tumor types from http://www.cbioportal.org/ as accessed on 9 January 2022.

All p53 protein family members have a domain structure and are expressed in isoforms. Two major types of isoforms are a result of the expression from two alternative promoters, P1 and P2. These two isoforms are the transcriptionally active (TA) isoforms, acting as tumor suppressors and the N-terminus truncated isoforms (dN), dominant-negative towards TA isoforms; acting as oncogenes. In cancer, the degree of methylation of P1 and P2 dictates the ratio between the TA and dN isoforms and has been reported to significantly alter the response to chemotherapy in several cancer types [3]. In addition, all p53 family proteins undergo alternative splicing at the C-terminus, as exemplified by p53 and p73 (Figure 1A). Various isoforms have been demonstrated to play different functions in cancer development and therapy resistance.

Our still expanding understanding of the biology of the p53 proteins divulges that there is a high structural and functional homology between all p53 family members, and p73, as p53, binds Mdm2 (Figure 1B), MdmX, and p300/CAF and activates several classical p53 promoters, including *CDKN*1A, *bax*, *mdm*2, *gadd*45, cyclin G, IGFBP3, 14–3–3σ [4]. The p73 protein has a high sequence identity with p53 and exists in cells as a constitutively open tetramer in a way that resembles the structure of wild-type p53. Akin to p53, p73 activity is controlled by protein concentration and p73 protein undergoes similar post-translational modifications as p53 [5]. The full-length p73 isoform (designated as TAp73α) recognizes the same range of p53 genes involved in apoptosis, cell cycle, or senescence, is an important tumor suppressor which compensates for p53 loss in tumor regression and can be reactivated pharmacologically using repurposed drugs, as I have discussed in [3].

P53 is the best-characterized protein among the p53 protein family and is known to possess both transcription-dependent and transcription-independent functions. P53 binds to the target DNA sequence through its centrally located DNA binding domain (DBD) and drives the activation or repression of gene expression. The mechanisms of p53-mediated gene activation are quite well understood, yet the mechanism of p53-mediated gene repression remains elusive.

In their work, S Peuget and G Selivanova [7] discussed the different mechanisms by which p53 regulates gene expression. According to the available information coming from the ChIPseq data, in cancer cells, p53 binds to target promoters via a universal pattern that seems to be independent of the stress stimuli applied. This pattern is composed of around 100 genes and comprises divergent biological functions, such as apoptosis, cell cycle arrest, cell differentiation, or DNA repair. Yet, the authors highlight that complexity is added to this pattern, since only a limited number of these genes are upregulated universally by p53 across all tissue types. Thus, as concluded by the authors, p53 activity at its target promoters is very much context-dependent. One of the plausible explanations for the observed pattern is that the p53-mediated transcriptional programs are tuned by the epigenetic state of the chromatin, which in turn affects the availability of p53-responsive elements (RE), by p53 post-translational modifications (PTMs), and by the range of co-regulators.

As much as our knowledge of p53-mediated gene transactivation is broad, our understanding of the mechanism of gene repression by p53 remains vague. p53 represses its target genes by means of a direct association with the promoter and by recruiting the co-repressors or outcompeting the co-activators. Of note, p53 was shown to act in concert with the Sp1 transcription factor to repress a number of metabolic genes in breast cancer cells [8] or to outcompete Sp1 in its target promoters such as hTERT proximal promoter [9].

Authors further highlight that p53 silences the expression of target genes through mediating alterations to the methylation state of chromatin, since p53 was shown to regulate the transcription of the DNA methyltransferases DNMT1, DNMT3a, and DNMT3b.

Meanwhile, recent evidence may suggest that p53 represses its target genes mainly via the upregulation of CDKN1A and consequent p21-mediated inhibition of CDK and activation of the repressor DREAM complex. However, these studies do not take into consideration the lineage- and stress-dependent responses. Thus, the above mechanism should likely not be considered as the major mechanism of p53-mediated gene repression. In addition, the available ChIPseq data also allow for concluding that in repressed genes, p53 has a short residence due to the transient binding mode of p53. Thus, p53-mediated activation and repression differ according to the p53 occupancy in the target genes. The authors explain that repression does not require keeping the chromatin in an open state, and hence the difference in the p53 occupancy between activated and repressed promoters. Apart from what is known about the regulation of transcription based on the gene silencing experiments and the expression data, little is yet known about the mechanisms of gene regulation by other p53 family members, namely p73 and p63. The development of high affinity, validated, ChIP-grade isoform-specific antibodies, allowing for potent binding to endogenously expressed proteins, will enable further progress in this field.

*TP*53 is one of the most frequently mutated genes in human cancers. Somatic mutations occur in about half of all cancer types, and germline *TP*53 gene alterations underly the rare genetic condition called Li–Fraumeni syndrome (LFS). LFS predisposes patients to the early or very early onset of tumors; patients have a high probability of developing at least one cancer during their lifetime and have around 40% probability of facing a second cancer diagnosis regardless of their age at the first cancer diagnosis [10]. Regardless of the type of cells affected by *TP*53 mutations, somatic or germline, the inactivation of p53 is associated with aggressive disease and poor outcomes in the majority of cancer types.

The majority of *TP*53 mutations in human cancers are of the missense type and are classified as driver mutations (Figure 1C). Missense mutations can be divided into two major groups, DNA contact mutants and structural mutants. The most frequent mutations in the *TP*53 gene occur in the DNA binding domain at the amino acid positions 175, 245, 248, 249, 273. Substitutions at these positions destabilize p53 and disrupt p53 tumor suppressor function [11]. The most abundant hot spot mutations in human cancers are the substitutions of arginine to histidine at positions 273 and 175, which induce structural change in the p53 DNA binding domain and render the protein partially (273) or fully inactive (175) as a tumor suppressor.

p53 is a zinc-binding protein and requires Zn^2+^ to retain the proper conformation of the DBD domain. The substitution of arginine 175 to histidine alters Zn^2+^ binding and unfolds loop 2 (L2) of p53 DBD, which is normally responsible for the binding to the minor groove in the DNA. All in all, missense mutation at 175 abolishes wild-type p53 ability to recognize its target genes involved in tumor suppression, yet promotes new functions, called gain-of-function (GOF), through direct or indirect associations with DNA.

Chiang et al. [12] highlighted that the R175H mutant gains new functions through several mechanisms. Firstly, p53-R175H, due to changes in the L2 region, might non-specifically bind to various DNA sequences which are different from the wild-type p53 responsive elements, and this transactivates a new set of target genes. Next, p53-R175H may also interact with the range of transcription factors to enhance or suppress the expression of their target genes. In addition, p53-R175H is prone to aggregation and may, via oligomerization with TA isoforms, inhibit wild-type p53 and other p53 protein family members, p73 and p63, which will be discussed in more detail below.

The network of cellular pathways affected by the mutant p53 protein is extensive, and we are still not close to comprehending all of them. The reason behind our ignorance is the high intrinsic instability of p53-R175H and the co-aggregation potential of the misfolded protein, which in turn enables an almost indefinite number of new protein–protein interactions in cancer cells.

In their work, the authors [12] discuss the impact of p53-R175H on the induction of genomic instability, the promotion of tumor cell growth, migration, invasion and metastasis. In example, the authors describe that p53-R175H induces chromosomal translocations by impairing the recruitment of MRN/ATM to DNA double strain break sites by interacting with Mre11 protein. Next, mutant p53 was demonstrated to act in concert with ETS2 transcription factor and transcriptionally upregulate a number of genes involved in chromatin remodeling, such as methyltransferases MLL1 (KMT2A), MLL2 (KMT2D), and acetyltransferase MOZ (KAT6A). Relevantly, an important link between p53-R175H and oncogenic Kras protein was recently described. p53-R175H induces the expression of hnRNPK, a splicing regulator, which in turn generates splicing isoforms of GTPase-activating proteins. The isoforms are inactive towards oncogenic Kras and contribute to uncontrolled tumor growth.

Growing evidence demonstrates that p53-R172H promotes the invasion of various tumor types through several mechanisms. In example, mutp53 was shown to bind to p73 and prevent its association with NF-YA, NF-YB, and NF-YC complex. This leads to the transactivation of platelet-derived growth factor receptor beta (PDGFRβ), a key factor in pancreatic cancer cell invasion. In addition, crosstalk between mutant p53 and SMAD2 protein, a member of the TGFβ pathway, leads to mutant p53-R175H phosphorylation, the inhibition of TAp63, and the promotion of metastasis. The authors furthermore describe other critical players involved in cancer cell metastasis and shown to be dependent on mutant p53-R175H, such as mutant EGFR, miR-130b, miR-142-3p, Twist1 or Rac1.

Besides the already listed biological functions, the gain-of-function mutant p53 also drives cancer cells’ stemness, the ability of self-renewal. For example, mutant p53-R175H indirectly stabilizes the YAP/TAZ complex and triggers the transcriptional activation of genes that promote cancer-stem-cell survival factors such as cysteine-rich angiogenic inducer 61 (CYR61), connective tissue growth factor (CTGF), baculoviral IAP repeat containing 5 (BIRC5) protein.

Lastly, among other cellular processes, mutant p53 was described to affect cancer cells’ energetics and to promote drug resistance. The GOF mutant proteins including p53-R175H confer resistance towards chemotherapy through the activation of the miR128-2/E2F5/p21 axis and the consequent inhibition of caspase 3 cleavage upon chemo treatment.

The mitochondrial citrate carrier, Slc25a1, appears to underly the resistance to platinum-based chemotherapy in certain tumor types. Chiang et al. describe that *SLC*25A1 gene is transcriptionally activated by mutant p53 and contributes to its GOF. Mutp53 additionally affects glycolysis and contributes to the so-called Warburg effect through inducing relocation of glucose transporter one, GLUT1, responsible for glucose uptake. Moreover, under glucose deprivation conditions, p53-R175H localizes to the cytoplasm and inhibits phosphorylation and activation of AMPK kinase, an important sensor of ATP/ADP ratio [12].

Since mutant p53 is inadvertently linked to aggressive disease and poor outcomes in human cancers, current efforts strive to target mutant p53 for improved cancer therapy. The most advanced mutant p53 reactivating molecule in Phase II and Phase III clinical development in cancers is APR-246/eprenetapopt. The active form of APR-246, methylene quinuclidinone (MQ), forms defined conjugates with cysteine 124, 229, and 277 in mutant p53 DBD domain [13,14], refolds it to wild-type conformation and induces apoptosis in mutant p53-loaded cancer cells. In addition, in their work, Chiang et al. discussed that arsenic trioxide, approved to treat acute promyelocytic leukemia, also targets cysteine residues in mutant p53, refolds it into its wild-type conformation and reactivates a range of structural mutant p53 proteins in cancer cells.

Another clinically advanced strategy to target mutant p53 protein in cancer cells discussed by authors is to promote its degradation. In cancer cells, mutant p53 is stabilized by HDAC6-mediated Hsp90 deacetylation and a resulting multi-protein mutant p53 stabilizing complex. Relevantly, the FDA-approved HDAC inhibitor suberoylanilide hydroxamic acid (SAHA/Vorinostat) downregulates mutant p53 through Mdm2-mediated ubiquitination and degradation and the combined treatment with Hsp90 inhibitors, 17DMAG or ganetespib delivered even better pre-clinical responses. SAHA is currently tested in a clinical trial in advanced *TP*53 mutant malignancies.

Since HDAC6/Hsp90-dependent mutant p53 accumulation relies on RhoA geranylgeranylation, repurposed statins, inhibitors of the mevalonate pathway, are currently tested in the clinical setting for their efficacy in *TP*53 mutant and wild-type malignancies.

In conclusion, the discussed articles published in this Special Issue cover a broad scope of the newest available information on the p53 protein family structure, function, and potential targeting for improved cancer therapy.
